# The genetics of resistance to infectious pancreatic necrosis virus in rainbow trout unveiled through survival and virus load data

**DOI:** 10.3389/fgene.2024.1484287

**Published:** 2024-11-19

**Authors:** Aqeel Ahmad, Muhammad Luqman Aslam, Øystein Evensen, Amr A. A. Gamil, Andreas Berge, Thoralf Solberg, Armin Otto Schmitt, Bjarne Gjerde

**Affiliations:** ^1^ Department of Breeding and Genetics, Nofima, Ås, Norway; ^2^ Department of Animal and Aquacultural Sciences, Norwegian University of Life Sciences, Ås, Norway; ^3^ Department of Paraclinical Sciences, Faculty of Veterinary Medicine, Norwegian University of Life Sciences, Ås, Norway; ^4^ Osland Genetics, AS, Norway; ^5^ Department of Animal Sciences and Center for Integrated Breeding Research, Georg August University, Göttingen, Germany

**Keywords:** IPNV, rainbow trout, challenge test, GWAS, virus load, QTLs, selective breeding

## Abstract

Infectious Pancreatic Necrosis virus (IPNV) is one of the major threats to the animal welfare and economy of the rainbow trout farming industry. Previous research has demonstrated significant genetic variation for resistance against IPNV. The main objective of the study was to investigate the genetic architecture of resistance against IPNV in rainbow trout fry. To achieve this, 610 rainbow trout fry, from a full factorial mating between 5 sires and 5 dams, were bath challenged with the IPNV isolate (IPNV-AS) from Atlantic salmon reared at a commercial farm. The resistance against IPNV was accessed using three different phenotypes; binary survival (BS), total days survived (TDS) and virus load (VL) recorded on the fish throughout the 40-day challenge test. All fish were genotyped using a 57K Affymetrix SNP array. The IPNV-AS isolate resulted in an overall mortality of 62.1%. The heritability estimates for survival (BS h^2^ = 0.21 ± 0.06, TDS h^2^ = 0.25 ± 0.07) and VL traits (h^2^ = 0.23 ± 0.08) were moderate and indicative of potential use of selection for increased resistance to IPNV in rainbow trout selective breeding programs. The unity estimated genetic correlation between the two survival traits (BS and TDS) indicates that the traits can be considered the same trait. In contrast, a moderate favourable negative genetic correlation was found between VL and the two survival traits (−0.61 ± 0.22 to −0.70 ± 0.19). The GWAS of the traits with many QTLs crossing the chromosome-wide Bonferroni corrected threshold indicates the polygenic nature of the studied traits. Most of the 10 possible identified genes were found to be linked with immunity or viral pathogenesis, which could be potentially responsible for the significant genetic variation in survival against the IPNV-AS. The QTL validation analysis revealed no significant difference in the mortalities and VL among the three genotypes of the detected QTL. The VL trait showed larger variation among the dead fry and with a concordant pattern with the two survival phenotypes, but with no significant difference in the proportion of IPNV VL positive samples in the dead and the survived fry. Overall, the results indicate the polygenic nature of the studied traits and support the use of genomic selection to improve resistance against IPNV in rainbow trout breeding companies.

## 1 Introduction

Infections and diseases pose the biggest threats to the Norwegian rainbow trout industry, with an annual production loss of 3 million grown-up rainbow trout (16% of all rainbow trout smolt stocked into net cages), in addition to massive juvenile production losses over the last two decades (Fiskeridirektoratet, 2024). Several contagious diseases cause these losses in many rainbow trout countries globally, including IPNV (Tapia et al., 2020), Infectious Hematopoietic Necrosis (IHN) (Balakhnina and Melnikov, 2024), Viral Hemorrhagic Septicemia (VHS) (Baillon et al., 2020), Flavobacteriosis (Bacterial Cold Water Disease) (Saticioglu et al., 2021). The global epidemiological studies documented the presence of different IPNV genotypes in many parts of Asia, Europe, America, and Africa (Ahmadivand et al., 2020; Mulei et al., 2018; Persson et al., 2023; Tamer et al., 2021; Ulloa-Stanojlovic et al., 2022; Zhu et al., 2017). In Norway, IPNV is ranked among the top five increasing problems for rainbow trout juveniles due to increased mortality, reduced welfare, and stunted growth (Fiskeridirektoratet, 2024).

IPNV is a significant threat to the salmonids, including rainbow trout, in both freshwater and seawater phases (Evensen and Santi, 2008). The field outbreaks at different fish farms can lead to 10%–100% mortality depending on the fish genetics, IPNV genogroup, and production environment including growth stage, stress, overcrowding, and husbandry (Bebak-Williams et al., 2002; Lapierre et al., 1986; Rønneseth et al., 2007). Scientific studies have confirmed both the horizontal transmission (among individuals of the same generation sharing the same space) as well as the vertical transmission (from parents to progeny) of IPNV (Kar, 2016). It remains prevalent in aquaculture for a prolonged time despite proper disinfection, effective management, and efforts to vaccinate the fish. The IPNV is highly transmissible, economically significant, and a genuine concern for rainbow trout farming.

The IPNV attacks the fish and multiplies within the host cell’s cytoplasm. The prime targets of the virus are the exocrine pancreas and the liver. The histopathological hallmarks of IPNV infection are the targeted destruction of exocrine pancreatic tissue and zymogen granules released from necrotic cells (Zhu et al., 2017). The pathological and clinical indications are darker skin colour, abdominal distension, abnormal swimming, sticky and trailing faeces, and stunted growth (Munang’andu et al., 2016).

The fate of an IPNV outbreak depends on the genotype of IPNV, the different genetics and immune capabilities of the fish, and environmental factors. Fish genetics is a tool to demonstrate variable resistance to IPNV in different fish reared under similar conditions and challenged with the same viral isolate (Dopazo, 2020; Houston et al., 2010; Ozaki et al., 2001). Field survival and challenge test data have documented the significant genetic variation for resistance against IPNV (Houston et al., 2010; Kjøglum et al., 2008; Storset et al., 2007; Wetten et al., 2007). A significant breakthrough was the discovery of major QTL associated with resistance against IPNV in Atlantic salmon, the selection for which has led to a sharp decrease in IPNV outbreaks in Norwegian salmon (Sommerset et al., 2020). However, the discovery of a new IPNV variant has posed a new challenge, as this variant can cause high mortality even in genetically selected Atlantic salmon (Hillestad et al., 2021). Scientific studies in rainbow trout have identified genetic markers with moderate effects, suggesting that the resistance against IPNV is mainly a polygenic trait (Ozaki et al., 2007; Ozaki et al., 2001; Rodriguez et al., 2019). This highlights the possibility of using genomic selection to combat IPNV infection.

The current research was designed with the aim to uncover the genetic architecture of resistance of rainbow trout to an IPNV-isolate from Atlantic salmon (IPNV-AS) through the study of I) the association between the survival (binary) phenotypes and the quantitative virus load phenotype, II) verification of the QTL for resistance against IPNV detected in a previous study, and III) the genetic variance and genomic regions potentially associated with the resistance against IPNV in rainbow trout.

## 2 Material and methods

### 2.1 The fish

The IPNV challenge test was performed with 610 rainbow trout fry belonging to 25 full sibs families and the 2021 year class of the Osland Genetics strain. The fish families were selected based on the genotypes of the most significant SNP “AX-89932,951” within the QTL region identified for resistance against IPNV by Aslam et al. (2019). Based on the genotype of the SNP, the parents of the fish were divided into three groups; IPNV-resistant (RR; having both copies of favourable allele), IPNV-heterozygous (RS; having one copy of favourable allele) and IPNV-susceptible (SS; having no copy of favourable allele). The planned mating design was a full factorial design with sires and dams from all three groups, as shown in Table 1. Unfortunately, due to human error, only four of the nine planned crosses were achieved (as indicated by cells shaded in grey in Table 1), leading to an unequal representation of the progeny families and high number of heterozygotes among the 610 rainbow trout fry challenged.

**TABLE 1:** Offspring genotypes (and no. of individuals, N) obtained from the mating of the three parental genotypes (RR = homozygous IPNV-resistant; RS = heterozygous susceptible; SS = homozygous IPNV-susceptible) of the top SNP in the selected QTL. Only four out of nine planned crosses were achieved and indicated by cells shaded in grey.

Sires/Dams (no. of parents)	RR (N = 4)	RS (N = 0)	SS (N = 1)
RR (N = 0)	RR	RR RS	RS
RS (N = 2)	**RR (N = 123)** **SR (N = 132)**	RS	**RS (N = 28)** **SS (N = 28)**
SS (N = 3)	**RS (N = 218)**	SR SS	**SS (N = 75)**

### 2.2 Isolation and characterization of IPNV isolate

The IPNV-AS isolate was initially isolated from kidney samples obtained from IPNV-infected Atlantic salmon in December 2016. Samples were homogenized in 10 mL of L15 medium supplemented with gentamicin using stomacher. After homogenization, homogenates were collected and cell debris was pelleted by centrifugation at 4°C. The cleared supernatant was filtered at 0.2 μm before being frozen (−80°C) until required. For virus isolation, 10 μL of the filtered homogenates were added to a 24-well plate of 80% confluent CHSE (chinook salmon embryo, p47) cells and harvested after the development of cytopathic effect (CPE) at 14 days. Cell debris was then pelleted by centrifugation as described above and kept at −80°C. This initial virus stock was then propagated in RTG2 fish cells.

To characterize the virus isolate, viral RNA was extracted from the infected culture fluid (ICF) using the QIAamp Viral RNA isolation kit (Qiagen). The extracted RNA was then used as a template to amplify the VP2 gene of the IPNV using the Qiagen One-step RT-PCR kit as well as the primers described by Gamil et al. (2016). The VP2 gene was then sequenced using a commercial service provider (Eurofins Genomics).

### 2.3 Challenge test

The 610 rainbow trout fry, with an average of 24 fry per family, were transported to VESO Vikan, Norway (https://www.veso.no/veso-aqualab) where they were bath challenged with the IPNV-AS in a single tank (0.6 m^3^). Before the challenge, the fry were allowed to acclimatize for 1 week according to the standard protocol with a stocking density of 40 kg/m^3^, 24-h exposure to light, and an average temperature of 12°C ± 1°C. Daily, the tank was cleaned and the fish were monitored closely to observe unexpected behaviour like loss of appetite. The fry were bath challenged with the IPNV-AS isolate at a concentration of 1 × 10^5^ – 5 × 10^5^ TCID_50_ (Median Tissue Culture Infectious Dose) per ml.

The challenge test lasted 40 days (May 25, 2021- July 8, 2021) until the day-wise mortality was <2 fish per day, after which the survivors were euthanized for sampling. A tissue sample was obtained from the tail of all the dead and survivors for DNA genotyping and parental assignment. A cross-sectional tissue sample from the middle of the 248 dead fry and all the 200 surviving fry was obtained for virus load (VL) analysis by real-time qPCR.

### 2.4 DNA genotyping

The tail tissue samples for DNA extraction were placed in Nunc-96 well plates filled with 99.5% absolute alcohol (free of water or fat) and were stored at 4°C before they were shipped to Nofima AS, Norway. The DNA genotyping was done by Identigen using a 57K Affymetrix Axiom SNP array (Palti et al., 2015). The initial filtration and quality control steps were done using the PLINK software (Purcell et al., 2007) to clean the genotyping data. Finally, 604 individuals and 38,433 SNPs, passed the quality check of minor allele frequency (MAF >2%), Hardy Weinberg equilibrium test (HWE *p*-value >10^−10^), and genotyping rate >98%.

### 2.5 RNA sampling

#### 2.5.1 RNA extraction and quantification

The cross-sectional tissue samples (back muscle) of the fry were collected in RNAlater vials and placed in 96-well plates marked with unique identifiers. RNA was extracted using the QIAGEN QIAsymphony RNA kit protocol (QIAGEN, 2024). The RNA quality and quantity were assessed using an Epoch microplate spectrophotometer. Purity was assessed using A260/A280 (RNA purity indicator parameter), and the final concentration of RNA in the sample was obtained in ng/µL. All the samples showed good purity and concentration of the RNA except for four, which were discarded; therefore, 444 samples were stored at −80°C until further RT-qPCR analysis.

#### 2.5.2 Real-time PCR analysis

The sequence of the primers used for targeting the structural VP2 gene of the IPNV and the reference (Beta-Actin) genes are presented in Table 2. Detailed information on the primers was already published by Gamil et al. (2016). The primers were ordered from Eurofins Genomics GmbH, Ebersberg, Germany. The BIORAD iTaq Universal SYBR Green one-step kit (Bio-Rad, 2022) was used to run the real-time qPCR analysis. The PCR mix contained 5 µL SYBR green, 0.25 µL reverse transcriptase, 0.5 µL of each primer, 0.875 µL nuclease-free water and 3 µL template to make a final reaction volume of 10 µL. All samples were placed in 96 well plates and run in duplicates for both the IPNV and reference Beta-actin gene. The following thermocycler profile was used for all PCR reactions: denaturation at 95°C for 10 seconds (s); annealing at 60°C for 39 s and finally elongation at 70°C for 10 s.

**TABLE 2:** Sequences of primers used for qPCR reactions. IPNV primers were used to target the VP2 region of the IPNV and Beta actin primers to target the reference housekeeping gene, the Beta actin.

Primer	5′- 3′ sequence
IPNV- Forward	CAA​CAG​GGT​TCG​ACA​AAC​CAT​AC
IPNV- Reverse	TTG​ACG​ATG​TCG​GCG​TTT​C
Beta Actin- Forward	CCA​GTC​CTG​CTC​ACT​GAG​GC
Beta Actin- Reverse	GGT​CTC​AAA​CAT​GAT​CTG​GGT​CA

Finally, the average cycle threshold (CT) value was calculated from the real-time qPCR output for all IPNV-positive samples. The CT values were then standardized across all PCR reactions using B-Actin, calibrator, positive control (IPNV isolate) and negative control (PCR master mix only). The following equation was used to calculate the relative VL in the studied samples.

$$Relative\;VL=\frac{\left[{\left(\;E_{ref}\right)}^{CT\left(Sample\right)}/{\left(\;E_{target}\right)}^{CT\left(Sample\right)}\right]}{\left[{\left(\;E_{ref}\right)}^{CT\left(Calibrator\right)}/{\left(\;E_{target}\right)}^{CT\left(Calibrator\right)}\right]}\times 1000$$

where E = primer efficiency; ref = reference Beta-actin gene; CT = threshold cycle for the test and calibrator samples.

### 2.6 Statistical analysis

The analysis of variance (ANOVA) was used to determine if there is any statistical difference in VL between the dead and the alive fry, and among three the genotypes of the identified QTL.

### 2.7 Variance component estimation

Estimates of variance and covariance components for the studied traits binary survival (BS), total days survived (TDS) and virus load (VL) were obtained from the following multitrait mixed linear model (MLM) (Equation 1), implemented in the GCTA package (Yang et al., 2011):

y=µ+Zu+e (1)

where y is a vector of *n* records for each of the traits, µ is the overall mean, Z is the incidence matrix and u is the vector of additive genetic effects. The vector of additive breeding values was assumed to follow $\tilde{u}MVN\left(0,A⊗G_{o}\right),$ where $u=\left(\begin{array}{c} u_{1}\\ u_{2} \end{array}\right),G_{o}=\left[\begin{array}{cc} \sigma_{u1}^{2} & \sigma_{u12}^{2}\\ \sigma_{u12}^{2} & \sigma_{u2}^{2} \end{array}\right]$ , G = genomic relationship matrix (GRM).

The (co)variance components were obtained using the restricted maximum likelihood (REML) approach in which the GRM was added to the random additive genetic polygenic part of the Mixed Model Equations.

The narrow sense heritability for each of the BS, TDS and VL traits was calculated as:

$$h^{2}=\frac{\sigma_{a}^{2}}{\sigma_{a}^{2}+\sigma_{e}^{2}}$$

where h^2^ = heritability, σ_a_^2^ = additive genetic variance and σ_e_^2^ = residual variance.

The genetic correlation between the traits was calculated as:

$$r_{g}=\frac{\sigma_{12}}{\sqrt{\left(\sigma_{1}^{2}\times \sigma_{2}^{2}\right)}}$$

where r_g_ = genetic correlation, σ_12_ = genetic covariance and σ^2^_1 or_ σ^2^_2_ = genetic variance of trait 1 and 2 in comparison.

### 2.8 Genome-wide association studies (GWAS)

The GWAS analysis was performed using the following single trait mixed linear model (Equation 2), implemented in the GCTA genomic tool package (Yang et al., 2014):

$$y=µ+\sum_{j=1}^{N=2}{pca}_{j}+M_{i}\alpha_{i}+Zg+e$$ (2)

where y = vector of phenotypes of the traits BS, TDS or VL, one at a time; µ = overall mean; pca = principal component; $M_{i}$ = incidence matrix for SNP i containing marker genotypes coded as AA=0,ABorBA=1,BB=2 ; $\alpha_{i}$ = allele substitution effect of SNP i ; Z = incidence matrix of genotyped fish, g = vector of genomic breeding values. The leaving-one-chromosome-out (--mlma-loco) approach was used for the association analysis of the trait. Afterwards, the GWAS summary was subjected to conditional analysis (--cojo-slct) to identify the independently and significantly associated SNPs.

The PLINK software (Purcell et al., 2007) was used to obtain the principal components (PCA) to account for population stratification and thus prevent false-positive genetic associations. The inflation factor (λ) was calculated using $\lambda =\frac{median\left(X^{2}\right)}{0.456}$ and qq-plots were generated to check any deviation of observed *p*-values from the expected distribution. To account for multiple testing, genome-wide (0.05/total no. of SNPs) and chromosome-wide (0.05/average no. of SNPs per chromosome) Bonferroni corrected significance thresholds were calculated to find the genome and chromosome-wide significant SNPs.

### 2.9 Gene mapping and enrichment analysis

All potential genes were searched within a ±20 kb region of the independent and significant QTLs with reference to the Omyk_1.0 (GCF_002163495.1) genome assembly. Gene Ontology (GO) (Harris et al., 2004) and Kyoto Encyclopedia of Genes and Genomes (KEGG) (Kanehisa et al., 2021) enrichment analysis were performed using Shiny GO 0.80 (Ge et al., 2020).

## 3 Results

### 3.1 Survival and virus load

**FIGURE 1:**
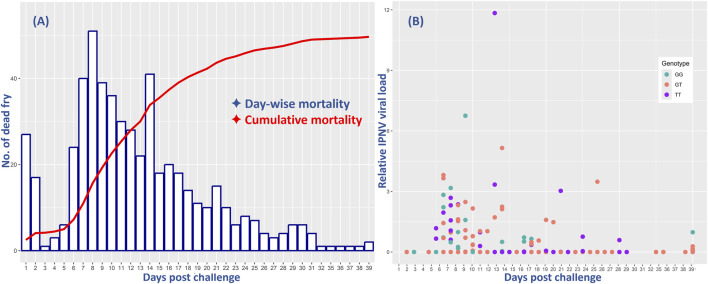
Comparative mortality of all and relative virus load (VL) of the 444 rainbow trout fry in the 40-day challenge test with the IPNV-AS. The *X*-axis represents the days post challenge and **(A)** shows the day-wise mortality of the fry (blue bars) and their cumulative mortality (red line), and **(B)** indicates the relative IPNV VL of the fry samples collected on different days. The three different colors of the dots correspond to the three distinct genotypes of the SNP selected for validation.

The survival analysis results showed a cumulative fry mortality of 62.1%. The day-to-day survival data showed a sharp increase in mortality from day 4, with the peak at day 8 followed by a slow decrease until the end of the challenge test (Figure 1). However, the relative VL showed that only 102 (23%) of all the 444 analyzed samples were positive with moderate to low levels of IPNV. There was no significant difference in the positive detection rate among the 375 fry that died during the test (18%) and among the 229 that survived the test (14%). However, ANOVA analysis shows that the relative VL was significantly higher in the tissue samples of the dead fish (mean VL = 0.53, SD = 1.3) than in those from the survived fish (mean VL = 0.008, SD = 0.07) (*p* < 0.001).

The day-wise mortality of all and the VL of the sampled 444 fish were found to be highest during the first 2 weeks of the challenge test, followed by a decrease both in mortality and VL until the termination of the test (Figure 1).

### 3.2 Characterization of IPNV isolate

The VP2 gene of the IPNV-AS carried the P_217_T_221_A_247_ motif that was different from the classical high virulent isolate NVI-015 of IPNV (T_217_A_221_T_247_) (Genbank ID: AY379740) in 11 amino acid positions, and in 9 amino acid positions from the low virulent IPNV isolate NVI-016 P_217_T_221_A_247_) (Genbank ID: AY379742) (Table 3).

**TABLE 3:** Comparison of the amino acids at distinct positions in the studied IPNV-AS isolate compared to the classical virulent IPNV isolate NVI-015 and the non-virulent IPNV isolate NVI-016.

Amino acid position	NVI-015 (AY379740)	IPNV-AS	NVI-016 (AY379742)
217	T	P	P
221	A	T	T
245	S	G	S
247	T	A	A
248	E	R	E
252	V	D	N
255	K	T	K
257	D	H	D
278	V	A	V
285	Y	H	Y
319	A	A	E
321	G	D	G
323	V	V	F

### 3.3 QTL validation

**FIGURE 2:**
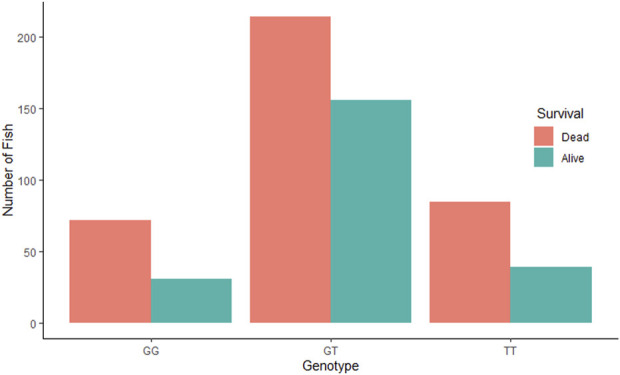
Comparative survival analysis of the three groups of rainbow trout fry based on the genotype of the tested SNP “AX-89932,951”. For each SNP-genotype, the orange bars show the total number of dead fry during the test and the green bars show the number of fry alive at the end of the test.

The QTL validation analysis yielded no significant association between the QTL detected by Aslam et al. (2019) and resistance to (against) IPNV. The bar plots show no significant difference in mortality across the three genotypes of the top SNP in the detected QTL (Figure 2). In addition, ANOVA revealed no significant difference in VL across the three genotypes (*p* > 0.05).

### 3.4 Genetic parameters

**FIGURE 3:**
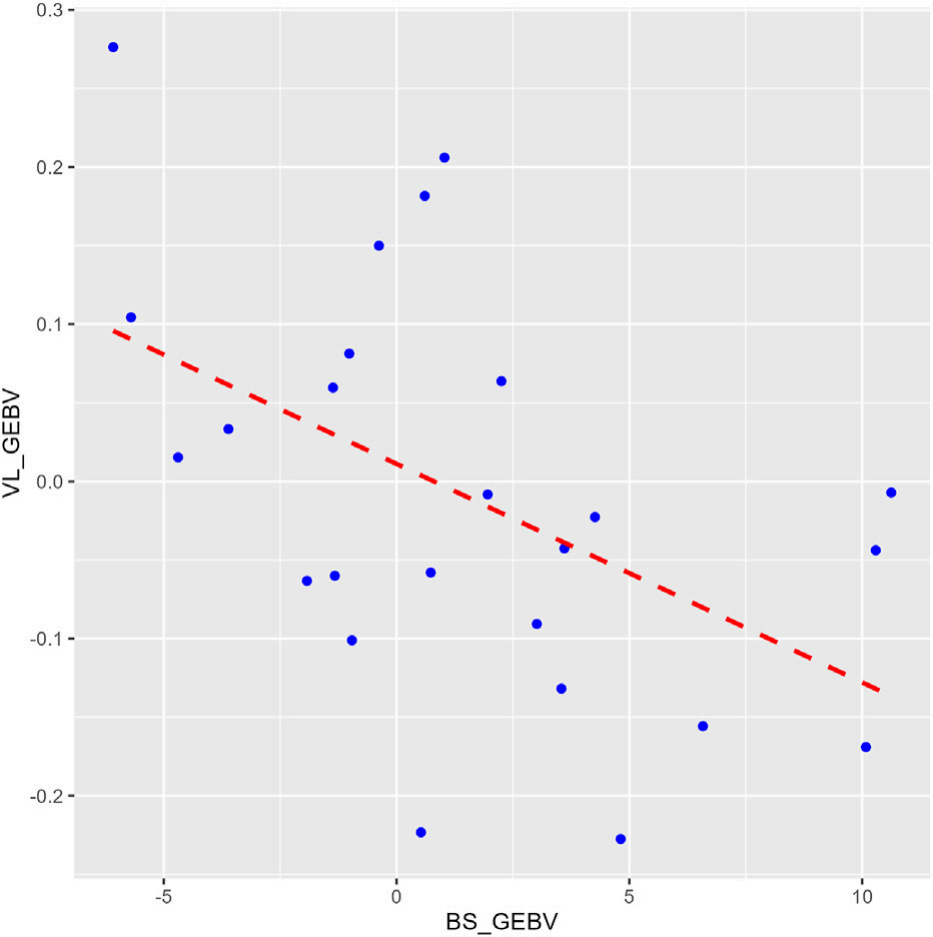
A plot comparing the family means of the genomic estimated breeding values (GEBVs) for the binary survival (BS_GEBV) and the virus load (VL_GEBV) trait. The *X*-axis represents the BS_GEBV and *Y*-axis represents the VL_GEBV. The red dashed line is the regression line of VL_GEBV on BS_GEBV.

The heritability estimate for IPNV VL (h^2^ = 0.23 ± 0.08) was similar to those for BS (h^2^ = 0.21 ± 0.06) and TDS (h^2^ = 0.25 ± 0.07). The two survival traits showed a unity genetic correlation (0.99 ± 0.01). In contrast, the genetic correlations of VL with each of the two survival traits were moderately negative (−0.61 ± 0.22 and −0.70 ± 0.19) (*p*-value = 0.01) but favourable as the lower the VL, the higher the survival, and *vice versa*. This correlation is also seen by the regression line (b = −17.6 ± 6.4) of the GEBVs family means of BS on VL (Figure 3).

### 3.5 GWAS for IPNV resistance

**FIGURE 4:**
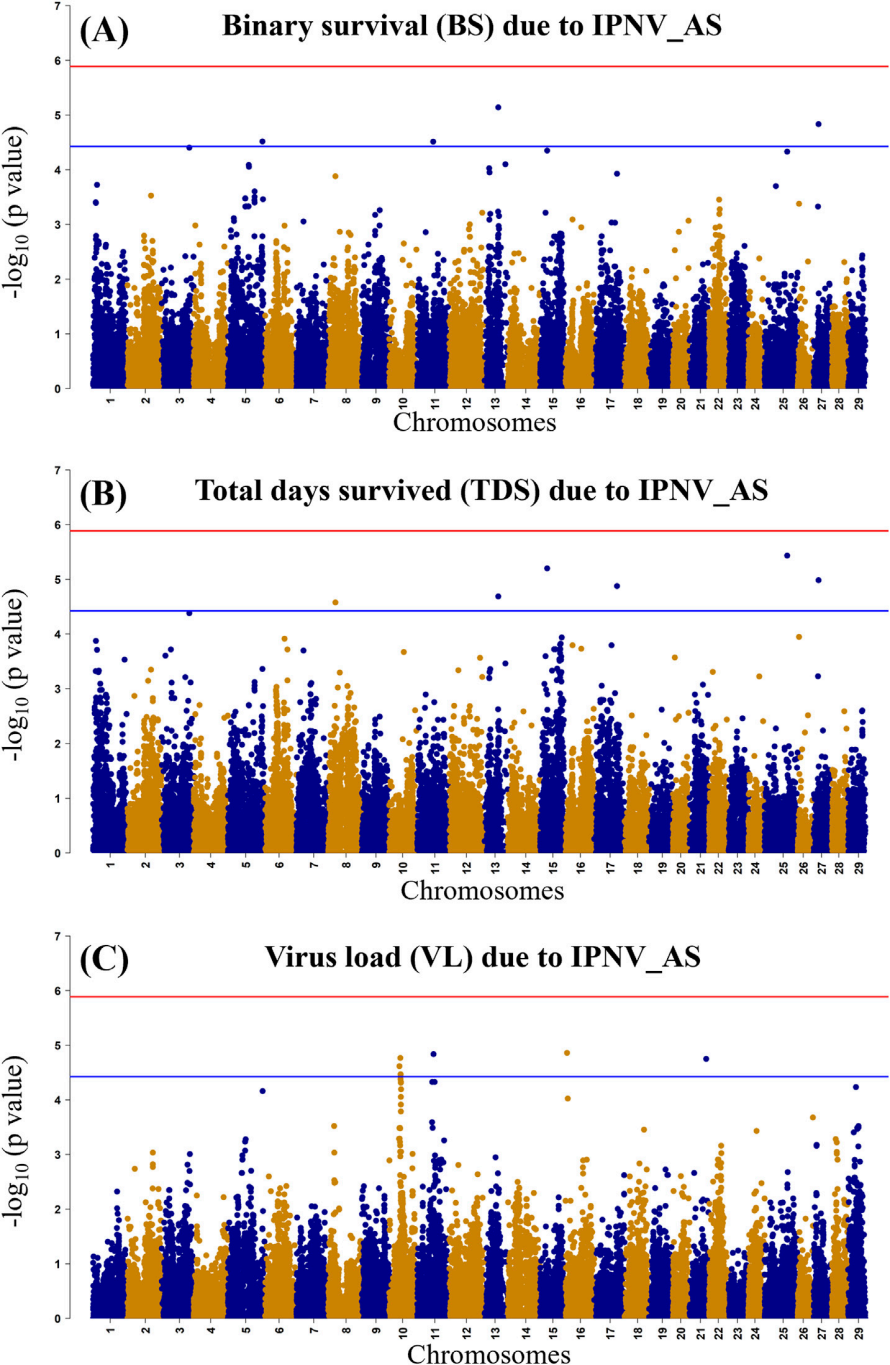
Manhattan plot of −log10 *p*-values derived from the genome-wide association studies (GWAS) of the BS trait **(A)**, the TDS trait **(B)** and the VL trait **(C)** from the rainbow-trout challenge test with IPNV-AS. The red and the blue lines represent the genome-wide Bonferroni significance threshold (−log10 *p*-value of 5.80 (*p* = 1.8 × 10^−6^)) and chromosome-wide significant threshold (−log10 *p*-value of 4.34 (*p* = 4.6 × 10^−5^), respectively.

The GWAS of fry survival and their VL from the challenge test with IPNV-AS showed sixteen SNPs crossing the chromosome-wide significant threshold but not the genome-wide threshold (Figure 4). The GWAS results suggest that the trait to a large degree is polygenic and the top SNP therefore contribute only to a small fraction of the genetic variance. A more powerful study design is required to detect true SNP effects. The conditional analysis of the GWAS summary resulted in 13 independent and significant SNPs (4 for BS, 5 for TDS, and 4 for VL) (Table 4). The inflation factor (λ) for the VL trait (λ = 1.03) shows that *p*-values are not inflated due to unknown reasons (e.g., population structure), while for both the BS (λ = 1.21) and the TDS (λ = 1.25) traits the *p*-values were moderately inflated. When the population structure was accounted for (see 2.7), the λ -values were reduced to 1.14 (BS) and 1.19 (TDS).

**TABLE 4:** The top and independent SNPs identified for the three studied traits (BS, TDS, and VL). Genomic positions of the SNPs on different chromosomes are shown in Mb.

Trait	Chr	SNP	Genomic position (Mb)	A1/A2	MAF	α	SE	p
BS	5	AX-89973557	85.60	A/G	0.37	0.13	0.03	3.03 × 10^−05^
	27	AX-89921954	12.53	T/C	0.40	−0.17	0.04	1.46 × 10^−05^
	11	AX-89945427	39.42	G/A	0.43	−0.15	0.03	3.07 × 10^−05^
	13	AX-89946108	40.18	G/T	0.35	0.17	0.04	7.22 × 10^−06^
TDS	25	AX-89917441	56.14	G/A	0.26	6.78	1.46	3.68 × 10^−06^
	27	AX-89921954	12.54	T/C	0.40	−4.97	1.13	1.04 × 10^−05^
	8	AX-89933013	18.09	T/G	0.27	5.90	1.40	2.65 × 10^−05^
	13	AX-89946108	40.18	G/T	0.35	4.67	1.10	2.06 × 10^−05^
	15	AX-89948367	17.66	G/T	0.14	9.25	2.05	6.33 × 10^−06^
VL	10	AX-89958047	27.74	G/A	0.07	0.68	0.16	1.71 × 10^−05^
	16	AX-89961019	3.53	A/G	0.04	0.84	0.19	1.38 × 10^−05^
	21	AX-89966517	43.87	C/A	0.03	0.83	0.19	1.78 × 10^−05^
	11	AX-89975610	40.11	C/T	0.11	0.54	0.12	1.45 × 10^−05^

Chr = Chromosome number; A1 = minor allele; A2 = major allele; α = Allele substitution effect; SE, standard error, p = raw *p*-value; BS, binary survival; TDS, total days survived; VL, virus load.

### 3.6 Identification of possible candidate genes

A total of 10 possible candidate genes (4 for BS, 4 for TDS, and 2 for VL) were detected within the vicinity of the significant and independent QTLs (Table 5). Some of the identified genes were found to have specialized functions related to viral infection and pathogenesis (*ushbp1, psma6, Fam172a*), inflammation and immune response (*Emp1*, *nr2f6)* and mitotic processes and abnormalities (*Fam184a*), while *Auts2* is sharing a peptide with the RUBV virus. The GO analysis showed that the identified genes are enriched with RNA processing/splicing pathways.

**TABLE 5:** Summary of potential genes found within ±20 Kb of the significant and independent QTLs identified for the studied BS, TDS and VL traits.

Trait	Chr	SNP	Annotation	Genes within ±20 Kb	Gene functions	References
BS	5	AX-89973557	5′UTR	*nr2f6*	This gene is involved in the survival and homeostasis of peritoneal B cells, a key mediator of humoral immunity. It also plays a role in suppressing T follicular helper cell that helps B cells develop long-term humoral immunity	Olson et al. (2019), Olson et al. (2022)
			3′UTR	*ushbp1*	Protein network component harmonin binding protein 1 is linked with Dengue viral infection and hepatocellular carcinoma (HCC) progression in humans	Krishnan et al. (2021), Zhang et al. (2020)
	27	AX-89921954	5′UTR	*psma6*	The proteasome subunit alpha type 6 gene controls the expression of interferon-stimulated gene (ISG15) that plays a crucial role in the pathogenesis of hepatitis C viral infection	Broering et al. (2016)
	11	AX-89945427	Intron	*Fam172a*	A family with sequence similarity 172 member A (fam172a) gene regulates cell growth, inhibits cell apoptosis and is involved in human epithelial to mesenchymal transition (EMT) It has demonstrated an effect against human cytomegalovirus (HCMV) as well	Chen et al. (2020), Li et al. (2010), Li et al. (2020)
TDS	13	AX-89946108	Exon	*Emp1*	Epithelial membrane protein 1 (EMP1) is a part of the NF-κB cascade that regulates inflammation, survival, and immune response and has implications for viral infections It is also involved in cancer cell proliferation, invasion, and metastasis	Liu et al. (2020), Yang et al. (2010)
			5′UTR	*Gsg1l*	Germ cell-specific gene 1-like protein (GSG1L) assembles either as a sole auxiliary subunit or co-assembles with transmembrane AMPA receptor regulatory proteins and thoroughly regulates the plasticity and strength of the glutamatergic synapse	Perozzo et al. (2023)
	8	AX-89933013	Intron	*Fam184a*	The Fam184a was found to be involved in mitotic processes and abnormalities It was classified as a risk prognostic gene in an endometrial cancer study	Golubeva et al. (2020), Liu et al. (2019)
	15	AX-89948367	Intron	*Myh6*	Myosin heavy chain 6 (MYH6) was closely related to ischaemic cardiomyopathy and heart failure in differentially expressed genes study MYH6 peptide is identical to the replicase polyprotein of the SARS-CoV-2 proteome	Anand et al. (2022), Chen et al. (2021)
VL	10	AX-89958047	3′UTR	*Auts2*	Autism susceptibility candidate 2 (AUTS2) was found to have a shared peptide with the RUBV virus In addition, the AUTS2 gene was found to play a key role in the transcription regulation of neurodevelopment, and homeostasis of excitatory synapses is crucial for neuropsychological disorders	Hori et al. (2021), Kanduc and Polito (2018)
	11	AX-89975610	3′UTR	ENSOMYG00000007399	A novel protein-coding gene with unknown function	

## 4 Discussion

IPNV is one of the most virulent and recurrent viral threats to economically important aquaculture species, including rainbow trout. It causes higher mortalities and stunted growth, leading to high economic losses and welfare concerns. Significant differences have been reported for the mortality of rainbow trout (Eriksson-Kallio et al., 2020; Tapia et al., 2020; Wetten et al., 2011) infected with different IPNV isolates in challenge tests.

### 4.1 IPNV challenge test

In the performed challenge test in this study, the IPNV-AS isolate was found to be virulent against rainbow trout fry, leading to peak mortality on day 8 post-challenge, followed by a slow decreasing slope until the termination of the challenge test at an overall mortality of 62.1%. These findings are consistent with Ozaki et al. (2007) who reported peak mortality on days 8 and 9 and a cumulative mortality of 36%–54%, and with those of Ahmadivand et al. (2020), with reported 40%–60% mortalities at 14 different Iranian rainbow trout farms with IPNV field outbreaks.

The mortality and VL were found to be highest during the first 2 weeks of the challenge test, followed by a decrease both in mortality and VL until the termination of the test. This associated pattern is in concordance with the results reported by Shao et al. (2022) who found high IPNV VL during the first phase of the IPNV i. p. injection challenge study with rainbow trout followed by low or no VL in different vital organs of the fish; but not with those of Tapia et al. (2020) who reported higher mortalities and low VL in rainbow trout after an IPNV genogroup 1 (type strain WB) challenge and comparatively higher VL and low mortalities after genogroup 5 (type strain Sp) infections.

The comparative assessment of the day-wise mortality and VL has shown comparable progression patterns. However, VL is highly variable in the dead fry during the challenge test as well as in the surviving fish at the end of the experiment. In addition, the absence of VL in dead fry may indicate clearance of the virus by the host, or point to other cause(s) of mortality than IPNV. Therefore, in this study, VL seems not to be a better predictor of disease resistance than survival phenotypes.

### 4.2 Characterization of IPNV isolate

The IPNV isolates carrying the P_217_T_221_A_247_ motifs were found to be avirulent or less virulent (Holopainen et al., 2017; Panzarin et al., 2018). Interestingly, the IPNV isolate used in this study had the same non-virulent motif, P_217_T_221_A_247_, but still resulted in high mortality (62.1%). Our results are in line with Ahmadivand et al. (2020) who reported the characterization of the IPNV isolates from the IPNV outbreaks in five Iranian trout farms and revealed the same avirulent P_217_T_221_A_247_ motifs that resulted in an overall mortality of up to 60%. Our finding is therefore in support of the view that the virulence of IPNV isolates in rainbow trout does not strictly follow the amino acid sequence of this motif in the viral VP2 gene.

The isolate used in this study also differed from both the classical Atlantic salmon virulent T_217_A_221_T_247_ and avirulent P_217_T_221_A_247_ strains of IPNV in 11 and 10 amino acid positions of the protein derived from the VP2 gene, respectively and these differences have possibly contributed to the high virulence. It is worth mentioning that this T_217_A_221_T_247_ isolate may also cause high mortalities in Atlantic salmon (Santi et al., 2004) suggesting that the changes observed in amino acids may have resulted in a better ability to infect and replicate in both species. The structural changes induced by the change observed in these amino acids warrant further investigation.

### 4.3 Estimates of genomic parameters

The magnitude of the heritability estimates for the studied traits were similar (BS: h^2^ = 0.21 ± 0.06, TDS: h^2^ = 0.25 ± 0.07 and VL: h^2^ = 0.23 ± 0.08) and thus consistent with three other rainbow trout challenge studies with IPNV which reported heritability estimates of h^2^ = 0.39 ± 0.08 (Flores-Mara et al., 2017), h^2^ = 0.24 ± 0.04 (Yoshida et al., 2019) and h^2^ = 0.30 ± 0.03 (Wetten et al., 2011). However, in an IPNV study with rainbow trout fingerlings, a very high heritability estimate for BS (0.83 ± 0.03) and a moderate heritability for time to death (0.53 ± 0.05) was found (Rodriguez et al., 2019). The latter high estimate for BS is from an i. p. challenge test and most likely obtained on the liability scale and therefore not comparable with the above binary heritability estimates from bath challenge tests.

The unity estimated genetic correlation between the two survival traits (BS and TDS) in this study shows that any of the two can be used as a selection criterion for increased resistance to IPNV in rainbow trout fry. The moderate favourable genetic correlation of VL with the two survival traits (−0.61 ± 0.22 to −0.70 ± 0.19) implies that selection for increased resistance to VL will result in a favourable correlated response in survival of fry to IPNV-AS but at a lower magnitude than when performing direct selection for increased fry survival.

### 4.4 QTL validation

Our validation study failed to detect any association between the studied IPNV-resistance traits and the QTL identified by Aslam et al. (2019). No significant difference (*p* > 0.05) was found between the three genotypes of the top significant SNP of the detected QTL for mortality and VL in the current population. The absence of genetic association can be attributed to several factors that are common in QTL studies. First, the GWAS results suggest that the traits are either polygenic in nature or the experimental design may not be strong enough to detect QTLs. Second, population specific genetic background could be the reason for the lack of replication in the current population due to differences in e.g., allele frequency, higher proportion of heterozygotes and linkage disequilibrium (LD). Third, the SNP identified in previous studies may be in LD with functional variants and the strength of their association could be different in the current population.

### 4.5 GWAS and post-GWAS

Genome-wide association analysis (GWAS) results indicate the polygenic nature of the resistance against IPNV, with many suggestive QTLs for the studied survival and VL traits. Previous studies have documented the identification of multiple QTLs for resistance against IPNV in rainbow trout (Ozaki et al., 2001; Ozaki et al., 2007; Rodriguez et al., 2019), however, we did not see any concordance between the QTLs reported in these studies and the suggestive QTLs detected in the current study. We found small signals for the BS and TSD traits on chromosomes 5, 8, 11, 13, 15, 17, 25 and 27 and small signals on chromosomes 10, 11, and 16 for the VL trait. There was however no clear concordance of the possible QTL for the survival and VL trait on these chromosomes. However, BS and TDS showed similar signals on Chr 13 and 27, whereas both the BS and VL showed a signal on chromosome 13.

The gene annotation analysis for BS revealed four potential genes within the (±20 Kb) genomic region of the significant and independent SNPs. However, our results for BS and TDS are not consistent with the genes that were identified in another GWAS study (Rodriguez et al., 2019) and a transcriptomic response study against IPNV in rainbow trout (Tapia et al., 2022). We identified Nr2f6 and ushbp1 genes in the 5′UTR and 3′UTR of the top SNP on Chr 5, which were found to be involved in humoral immunity and viral infection and could be important for IPNV pathogenesis and resistance. In addition, the Psma6 gene present on Chr 27 has been reported as a key regulator of the immune response gene against viral infection and was also found in a hepatitis C viral infection study. Additionally, gene fam72a on Chr 11 was found to regulate cellular growth, inhibit apoptosis, and protect against the HCMY virus. The QTL on Chr 13 was found to be in the exonic region of the emp1 gene, which is the part of NF-κB cascade involved in immune response and inflammation.

The TDS trait showed unique significant signals on Chr 8 and 15, in addition to signals on Chr 13 and 27 similar to the highly genetically correlated BS trait. The SNP on Chr 8 was found in the intronic region of the fam184a gene, which plays a crucial role in mitotic processes and abnormalities. In contrast, the myh6 gene on Chr 15 was found to have a peptide identical to the replicase polyprotein of the SARS-CoV-2 proteome. The significant SNPs on Chr 10 for the VL trait have shown the auts2 gene in 3′UTR, which is reported to have a shared peptide with the RUBV virus and also plays a major role in neurodevelopment. The significant signal on Chr 11 showed one protein-coding gene (ENSOMYG00000007399) that does not have a discovered function yet. In all, most of the identified genes are predominantly linked with immunity or viral pathogenesis, which could be potentially responsible for the significant genetic variation in survival against the IPNV-AS in the challenge test. Additionally, the GO enrichment analysis revealed that most of the identified genes were associated with RNA processing/splicing pathways. Previous studies have shown the central role of RNA splicing in host-virus interaction, IPNV pathogenesis, and possibly host defense against it Chauhan et al. (2019), Fukuhara et al. (2006). However, further QTL validation and gene expression studies are recommended better to understand the precise role of the identified genes in IPNV pathogenesis and resistance.

Overall, this study has suggested clues into the genomic basis of the survival of rainbow trout fry against IPNV-AS infection in a challenge test. However, the present study had some limitations as the number of tested fish was relatively low and with very few IPNV-positive samples. Additionally, the human error when crossing parents led to high heterozygosity in the progeny as only four of the nine planned crosses, which most likely resulted in an experiment with substantially less statistical power.

## 5 Conclusion

The moderate heritability estimates for the survival and virus load traits confirm the presence of significant genetic variance in both traits. GWAS have indicated the polygenic nature of the traits, with identified genes near significant loci being involved in immunity or viral pathogenesis. These indicative results support the use of genomic selection to improve resistance against IPNV infection for the benefit of rainbow trout breeding companies and their customers.

## Data Availability

The nucleotide sequence of the VP2 gene of the IPNV isolate used in this study is publicly available at the GenBank website (https://www.ncbi.nlm.nih.gov/genbank/) under the accession no. BankIt2891117 IPNV-L5 PQ584437. Additional data supporting the findings of the current study is available upon request.
